# Hydrogen Diffusion in Nickel Superalloys: Electrochemical Permeation Study and Computational AI Predictive Modeling

**DOI:** 10.3390/ma16206622

**Published:** 2023-10-10

**Authors:** Alfonso Monzamodeth Román-Sedano, Bernardo Campillo, Julio C. Villalobos, Fermín Castillo, Osvaldo Flores

**Affiliations:** 1 Facultad de Química, Universidad Nacional Autónoma de México, Ciudad de México CP 04510, Mexico; bci@icf.unam.mx (B.C.);; 2 Instituto de Ciencias Físicas, Universidad Nacional Autónoma de México, Cuernavaca CP 62210, Mexico; 3 Tecnológico Nacional de México/I.T. Morelia, Av. Tecnológico, No. 1500, Col. Lomas de Santiaguito, Morelia CP 58120, Mexico

**Keywords:** hydrogen diffusion, artificial neuronal networks, superalloys, electrochemical double cell, Garson’s algorithm

## Abstract

Ni-based superalloys are materials utilized in high-performance services that demand excellent corrosion resistance and mechanical properties. Its usages can include fuel storage, gas turbines, petrochemistry, and nuclear reactor components, among others. On the other hand, hydrogen (H), in contact with metallic materials, can cause a phenomenon known as hydrogen embrittlement (HE), and its study related to the superalloys is fundamental. This is related to the analysis of the solubility, diffusivity, and permeability of H and its interaction with the bulk, second-phase particles, grain boundaries, precipitates, and dislocation networks. The aim of this work was mainly to study the effect of chromium (Cr) content on H diffusivity in Ni-based superalloys; additionally, the development of predictive models using artificial intelligence. For this purpose, the permeability test was employed based on the double cell experiment proposed by Devanathan–Stachurski, obtaining the effective diffusion coefficient (D_eff_), steady-state flux (J_ss_), and the trap density (N_T_) for the commercial and experimentally designed and manufactured Ni-based superalloys. The material was characterized with energy-dispersed X-ray spectroscopy (EDS), atomic absorption, CHNS/O chemical analysis, X-ray diffraction (XRD), brightfield optical microscopy (OM), and scanning electron microscopy (SEM). On the other hand, predictive models were developed employing artificial neural networks (ANNs) using experimental results as a database. Furthermore, the relative importance of the main parameters related to the H diffusion was calculated. The D_eff_, J_ss_, and N_T_ achieved showed relatively higher values considering those reported for Ni alloys and were found in the following orders of magnitude: [1 × 10^−8^, 1 × 10^−11^ m^2^/s], [1 × 10^−5^, 9 × 10^−7^ mol/cm^2^s], and [7 × 10^25^ traps/m^3^], respectively. Regarding the predictive models, linear correlation coefficients of 0.96 and 0.80 were reached, corresponding to the D_eff_ and J_ss_. Due to the results obtained, it was suitable to dismiss the effect of Cr in solid solution on the H diffusion. Finally, the predictive models developed can be considered for the estimation of D_eff_ and J_ss_ as functions of the characterized features.

## 1. Introduction

Superalloys are materials used in applications that demand excellent corrosion resistance, creep, and good mechanical properties at high temperatures in aggressive media. These are employed in several applications such as petrochemical processes, transportation and gas industry, gas turbine components, and pressurized water reactors, among others [1,2,3,4,5,6]. The predominant base elements of the superalloys are nickel (Ni), titanium (Ti), iron–nickel (Fe-Ni), and cobalt (Co), and additionally, other elements are added to provide different properties to the material. For instance, as solid solution strengtheners, Mo, Cr, and W, for second phase particle formation, Ti, Nb, and Al, and for surface protection, Cr, Ti, and Y are utilized. The alloys in the Ni-Cr-Fe-C system are metals with a face-centered cubic crystalline structure (FCC), and throughout the gamma phase (γ) of the matrix, Cr carbides precipitate. Furthermore, in the case of Ti addition, the γ’ phase (Ni_3_Ti) is formed in a cube–cube relation with the matrix [1]. On the other hand, in metals, the H can diffuse and partially dissolve through the alloy matrix, causing detrimental effects; this has been widely studied under different frameworks, involving mathematical modeling, numerical methods, atomistic simulation, and experimental assessments [7,8,9,10,11,12,13,14,15,16,17,18,19,20,21,22,23,24,25]. The exposure to H can cause corrosion failure, due to a phenomenon known as hydrogen embrittlement (HE). Regarding the above-stated phenomenon, several mechanisms have been proposed to explain the effect of HE that generates within the materials. Some of the recognized proposals are as follows: “HELP”, Hydrogen Enhanced Local Plasticity, which establishes that embrittlement occurs due to the local mobility of dislocations, caused by the H dissolved in the matrix. Thus, inducing local plasticity, and, although it can be related to possible ductile behavior, the resultant fractures are brittle. The “HEDE” mechanism, Hydrogen Enhanced Decohesion Energy, postulates an expansion of the matrix generated by the H present, and this causes a decrease in the cohesion energy between atoms, causing possible fractures with lower energy [26,27,28,29,30]. Considering the experimental assessment of HE, it is possible to obtain the effective diffusion coefficient (D_eff_) and steady-state flux (J_ss_) with electrochemical tests. In addition, the entire characterization techniques are necessary to relate the effects of H on different materials. For this purpose, the double-cell experiment developed by Devanathan–Stachurski can be employed, and this method has been utilized to measure the amount of H that diffuses through materials. This evaluation technique is composed of two principal cells, the cathodic charging cell and the anodic detection cell [12,13,14,15,31]. The method concludes by obtaining current density (mA) vs. time (t) curves, to subsequently apply different models to calculate the D_eff_ and J_ss_. Regarding the above, due to the presence of defects, second-phase particles, and in general, the microstructural state of the superalloy, the D_eff_ and J_ss_, can be complemented with the trap density calculation (N_T_), obtaining values for reversible and irreversible sites. Thus, several models have been proposed, such as the McNabb–Foster, Oriani, and Dong et al., among others [23,24,32,33,34,35]. On the other hand, combining results of the material characterization techniques, H permeability evaluations, and mathematical modeling for H trapping, the application of artificial intelligence can be an excellent choice to develop a useful predictive model. Artificial neural networks (ANNs) are models able to predict behavior based on various characteristics, which do not necessarily have to be linear. Its application in science, technology, and engineering fields is increasingly frequent due to its powerful learning capacity [36,37]. In this work, the D_eff_, J_ss_, and N_T_ of experimental and commercial Ni-based superalloys were obtained employing the Devanathan–Stachurski double cell experiment. Furthermore, a predictive model using artificial neural networks was developed. The H permeability tests were applied on three types of superalloys previously designed and manufactured at different Cr contents, and on commercially available Inconel 600 and Inconel 690 alloys. The material was characterized and studied using qualitative and quantitative elemental chemical analyses, X-ray diffraction, and metallography. The AI models were trained for the prediction of D_eff_ and J_ss_ utilizing feed-forward backpropagation artificial neural networks, considering 10 experimentally acquired input variables. Finally, the relative importance of the input variables was calculated using the Garson’s algorithm.

## 2. Materials and Methods

### 2.1. Thermodynamic Study of the Phases in Ni-Cr-Fe-C Alloys

**Figure 1:**
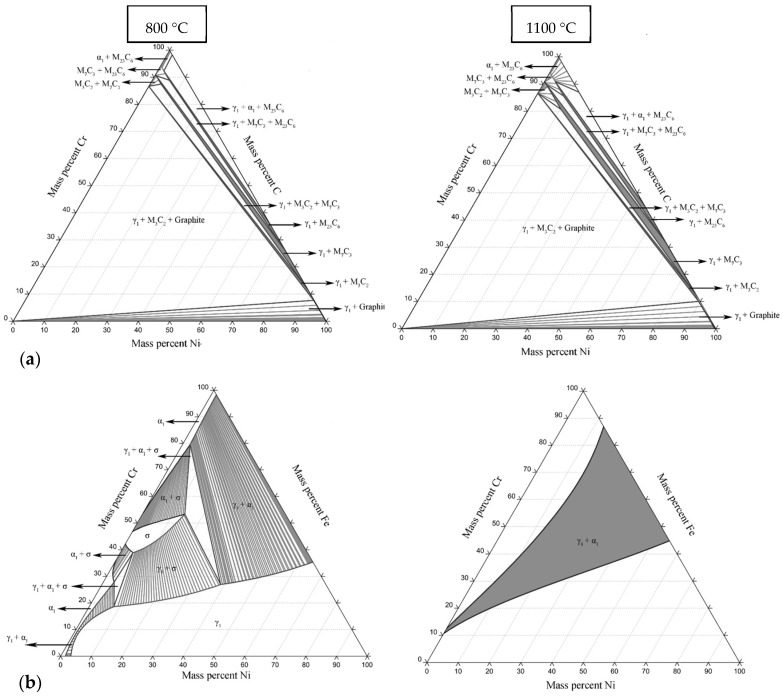
Ternary diagrams of (**a**) Ni-Cr-C and (**b**) Ni-Cr-Fe.

The system studied in this work comprises the Ni-Cr-Fe-C elements; consequently, the thermodynamically stable phase formation during the fusion and solidification processes was analyzed. The system is quaternary, complicating the calculation; nevertheless, the study can be separated into ternary systems. Before the fabrication of the experimental alloys, the thermodynamic analysis to achieve ternary phase diagrams was performed using the Calphad method (CALculation of PHAse Diagrams) at two different temperatures, 800 and 1100 °C. The basis of the thermodynamic analysis with the Calphad method can be described according to work reported by Wenliang Liu et al. 2020 [38], related to the main stable phases, which are as follows: “liquid, FCC, M_7_C_3_, and M_23_C_6_ ”. Figure 1 shows the ternary phase diagrams at a concentration of 28% Cr, 9% Fe, 0.5% C, and balanced by % Ni. Second-phase precipitates are formed in the Ni-Cr-C ternary diagram, principally the M_3_C_2_, M_7_C_3_, and M_23_C_6_ (see Figure 1a). The Ni-Cr-Fe system, as shown in Figure 1b, presented the formation of γ1 phase, and for the concentrations ≈30% Ni, ≈50% Cr, and ≈20% Fe, the γ1 + α1 is predominant. Also, the appearance of the σ phase is observed at high concentrations of Cr. This last phase has been widely reported experimentally and is characterized as detrimental, so it should be avoided in alloy design [39]. Therefore, based on the above, the possible phases in the quaternary alloys studied can be considered analogous to the Ni-Cr-C system. This is due to the high affinity of Cr to C; thus, the most common phases are γ1 + M_7_C_3_ + M_23_C_6_. In addition, in this work, the commercial alloy Inconel 690 was also tested; as a result, the γ’ phase (Ni_3_Ti) was found.

### 2.2. Material Acquisition, Manufacturing, and Characterization

Commercial alloys Inconel 600 and Inconel 690 (labeled as In 690 and In 600) were purchased in the 25.4 mm thick plate form. Regarding the experimental alloys, these were manufactured based on the Ni-Cr-Fe-C system described in the previous section, the process was achieved by employing an induction furnace with Ar atmosphere. The % Cr content was varied, having 28, 15, and 10% wt. (labeled as 28Cr, 15Cr, and 10Cr). The fusions were cast in recrystallized alumina metal mold, and subsequently, a hot-rolled process was applied at 950 °C, to form an equiaxed microstructure. Initially, sheet-form material was obtained with 6 mm of thickness, and later, it was hot-rolled in two steps, 6 mm → 3 mm and 3 mm → 1 mm; the commercial and experimental materials were analyzed and compared. On the other hand, elemental analysis using atomic absorption with coupled plasma source was applied to the experimental alloys. This was performed to verify their correct controlled elemental content during the fusion process; for this analysis, powder-filing of the alloys was prepared. Furthermore, an elemental CHNS/O analysis was employed, focused on the C content; the analyses were performed on the commercial and experimental alloys. Regarding the Inconel material, the chemical composition report was provided by the supplier; however, the CHNS/O study was also performed to confirm the C content. Table 1 shows the elemental content results, corresponding to the analysis of commercial and experimental superalloys. To analyze the possible influence of Cr in its solid solution state on the lattice of the experimental alloys (related to the lattice parameter), the X-ray diffraction (XRD) technique was achieved. Furthermore, this study complemented the assessment of the Ni-Cr-Fe-C alloy characteristic phases in an as-cast state. This evaluation was performed on a dendritic plate using XRD equipment with a coupled goniometer. Regarding the metallography, samples were prepared, starting with SiC sandpaper from 120 to 4000 grit, and then, 1 μm alumina coarse polishing, 0.5 μm and 0.03 μm alumina fine polishing, and finally, 0.01 μm cuboidal silica were used. The microstructure etching was fulfilled employing oxalic acid at 12% concentration and applying a potential of 15V with 1.5A current; the samples were observed using bright field optical microscopy (OM).

Subsequently, utilizing the ImageJ software, microstructure features, such as average grain size (AGS), average precipitate size (APS), and precipitated fraction (precipitation area/total area, P_A_/T_A_), were measured. The measurement methodology of all the samples is presented as follows:

- Average grain size (AGS, μm): Measurements of at least 50 grains applying the irregular polygons method. The evaluated area was ≈1 mm^2^ (up to 4 mm^2^ in microstructures with grain size greater than 150 μm).
- Average precipitate size (APS, μm) and precipitate fraction (P_A_/T_A_): The apparent precipitates were measured. The area analyzed for each sample was 4.3 mm^2^, and the number of measurements, as a function of the image quality, ranged from 150 to ≈3000 per sample, considering a roundness criterion > 0.5.

Finally, the samples were observed using scanning electron microscopy (SEM), the morphology of microstructures and precipitates were analyzed, and a qualitative elemental analysis was performed employing energy-dispersed X-ray spectroscopy (EDS).

### 2.3. Hydrogen Permeation Tests

**Figure 2:**
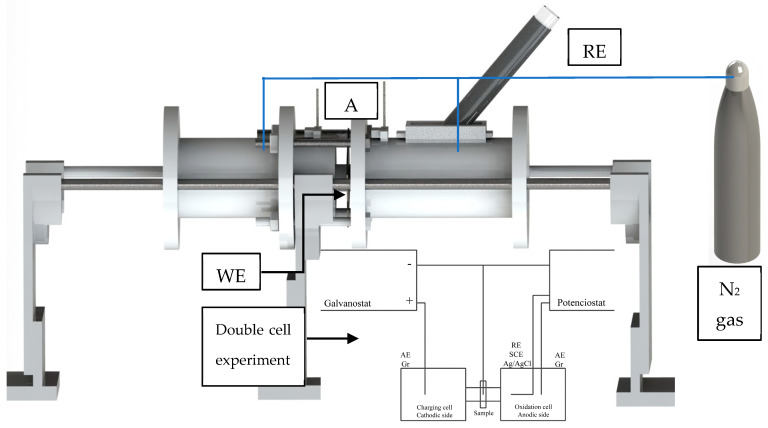
Schematic sketch of the double cell experiment. The auxiliary electrode (AE, graphite), reference electrode (RE, Ag/AgCl), and working electrode (WE, sample) are shown.

The H diffusivity of the Inconel and experimental material was assessed employing permeability tests, and additionally, it was performed in Inconel 600 with Au-Pd coating. The evaluations were carried out electrochemically using the experiment proposed by Devanathan and Stachursky, also stipulated in ISO 17081 [31]. This test consists of H charging on the sample surface, afterward, current measuring, and finally, the H diffusivity calculus from the obtained results. For this, the experiment comprises a double cell: the charging and H-detection cells. At the charging cell (cathodic side), H is generated using an acid solution; in this case, 0.1 mol/l of H_2_SO_4_ was aggregated. As a reaction retarder of the above-mentioned reaction, 5 × 10^−5^ mol/l of arsenic trioxide As_2_O_3_ was also added with the H → H_2_ recombination. Considering the detection cell (anodic side), an alkaline solution was used, and the added compound was 0.1 mol/l of NaOH. In addition, nitrogen gas was bubbled into the acid and alkaline solutions before and during each permeation test. On the other hand, the polarization control at the detection cell was carried out potentiostatically applying +300 mV. Finally, the H charging was performed at the cathodic side employing 40 mA/cm^2^ of current density. The double cell was designed and manufactured in polytetrafluoroethylene; the experiment scheme, with rendering of 3D CAD, is presented in Figure 2.

The samples for the permeability tests were prepared in a square shape, 25.4 mm per side. For this, the commercial alloys samples were manufactured by electrical discharge machining (EDM), obtaining sheets ≤ 1 mm of thickness. In contrast, the experimental alloy samples were obtained in the hot-rolled process described in Section 2.2 and later adjusted to required dimensions utilizing a diamond disc cutter. Regarding the test, the current (mA) vs. time (s) signals were obtained, and the resulting data were processed, achieving the transient analysis. Subsequently, Equation (1)–(4) were employed for the D_eff_ and J_ss_ calculus. The models presented in Equations (3) and (4) correspond to the Fourier and Laplace rise transient methods [40], respectively, and these were applied for comparison. Finally, to verify the fit of the experimental transients to Fick’s second law, Equation (5) was utilized, assessing *n* = 6. The Models (1), (2), and (5) have been explained in several works and are given by ISO 17081 [31].

(1) $$D_{eff}=\frac{L^{2}}{6t_{lag}}$$

(2) $$J_{\left(t\right)}=\frac{\frac{I_{\left(t\right)}}{A}}{F}$$

(3) $$\frac{J}{J_{ss}}=1-2exp\left(-\frac{\pi^{2}Dt}{L^{2}}\right)$$

(4) $$\frac{J}{J_{ss}}=\frac{2}{\pi^{1/2}}\frac{L}{{\left(Dt\right)}^{1/2}}exp\left(-\frac{L}{4Dt}\right)$$

(5) $$\frac{J(t)}{J_{ss}}=1+2\sum_{n=1}^{\infty }{\left(-1\right)}^{n}exp\left(-n^{2}\pi^{2}\tau \right)$$

### 2.4. AI Predictive Model

Once the experimental and commercial alloys were characterized, a predictive model was developed using artificial neural networks (ANNs). The processing methodology for inputs and outputs, as well as the training and evaluation procedures are presented below.

- Database: The database was set up from the results of elemental chemical characterizations, microstructural measurements, and the permeability test results. Furthermore, the results acquired and reported in previous work [5] were used to build the database. The resulting database was normalized from 0 to 1 and from −1 to 1; later, the sigmoid and hyperbolic functions, Equations (6) and (7), were implemented as transfer functions in the ANN neurons.
- Training process: Forty neural networks were trained, composed of one hidden layer and a linear transfer function at the output layer; this was implemented in the whole models. In contrast, the type of normalization data, transfer functions at hidden layers, and the number of neurons were varied. The training was supervised, and the learning was assessed by comparing the predicted with the experimental results using the method of minimum square error (MSE). Moreover, the Levenberg–Marquardt algorithm was also implemented for neural network learning; this has been widely used in this type of AI application [41,42,43]. On the other hand, the data for the training process were divided as follows: 70% for training, 15% for first validation, and 15% for final test. The last mentioned 15% was not part of the original code; this was performed to assess the predictive capability and verify the learning performance. The input and output variables of the predictive models used in the described processes are shown in Table 2.

(6) $$f\left(x\right)=\frac{1}{1+e^{-x}}$$

(7) $$f\left(x\right)=\frac{2}{1+e^{-2x}}-1$$

**Figure 3:**
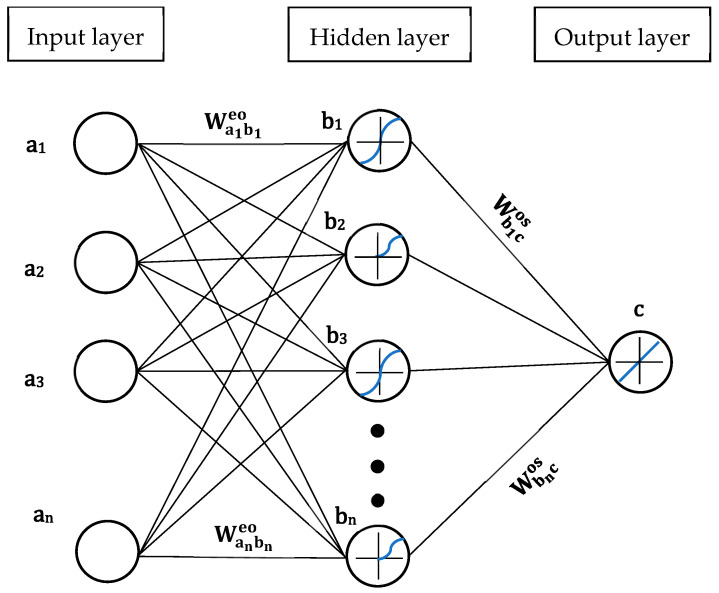
Schematic sketch of the ANN’s connections.

In addition, Garson’s algorithm [44] was applied using the trained ANNs, selecting the best models based on the correlation coefficient value. The algorithm was utilized to calculate the relative importance of each input variable on the output variables. The equation and a scheme example corresponding to this algorithm are presented below (see Equation (8) and Figure 3):

(8) $${IR}_{ac}=\frac{\sum_{b_{1}}^{N_{o}}\left[\frac{\left|W_{a_{n}b_{n}}^{eo}\right|\;*\;\left|W_{b_{n}c}^{os}\right|}{\sum_{a_{1}}^{N_{e}}\left|W_{a_{n}b_{n}}^{eo}\right|}\right]\;}{\sum_{a_{1}}^{N_{e}}\;\left[\sum_{b_{1}}^{N_{o}}\frac{\left|W_{a_{n}b_{n}}^{eo}\right|\;\times \;\left|W_{b_{n}c}^{os}\right|}{\sum_{a_{1}}^{N_{e}}\left|W_{a_{n}b_{n}}^{eo}\right|}\right]}$$

where *IR_ac_* is the relative importance of the variable “*a_n_*” in the input layer “*a*” over the output variable “*c*”; Ne and No depict the total number of input and hidden neurons, respectively. The weight of the connection is represented by *W*; the superscripts “*e*”, “*o*”, and “*s*” refer to input, hidden, and output neurons, respectively; finally, *bn* symbolizes the hidden layer and its neuron number. Figure 3 presents an artificial neural network arrangement related to the previous explanation of Garson’s algorithm equation.

## 3. Results

### 3.1. X-ray Diffraction

**Figure 4:**
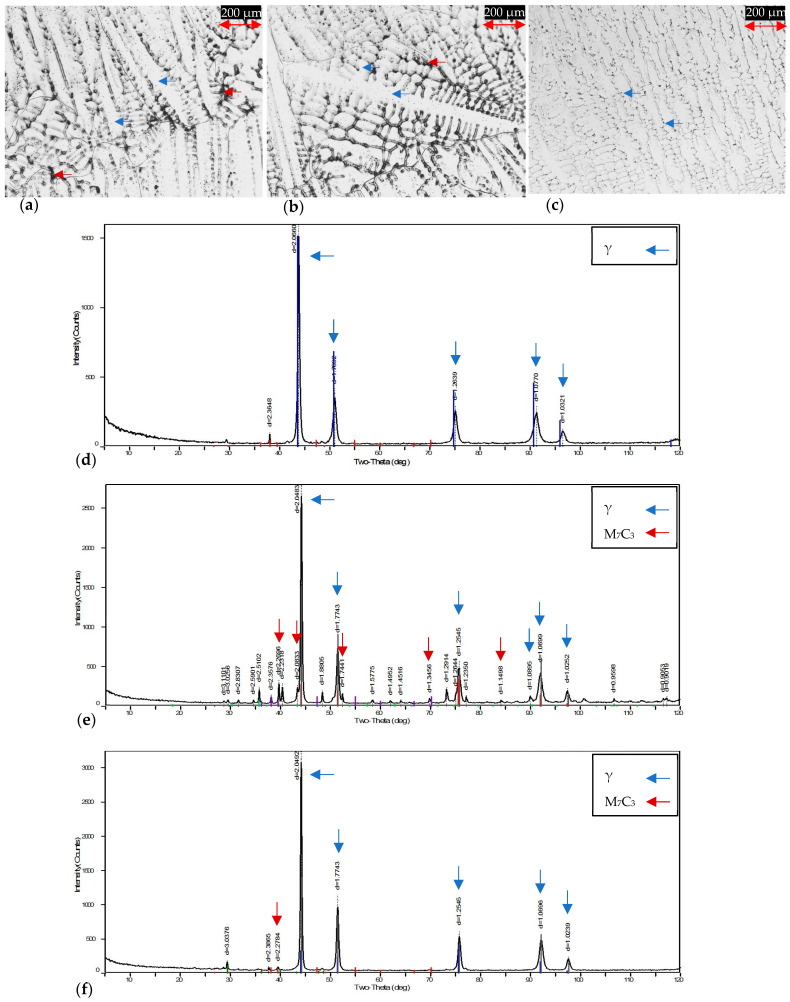
Dendritic microstructures and their corresponding XRD spectra: (**a**,**d**) 28Cr, (**b**,**e**) 15Cr, and (**c**,**f**) 10Cr.

Table 3 shows the XRD results of the experimental alloys. A slight increase in the interplanar spacing is observed for the 28Cr; this may be related to the Cr increment in the austenitic matrix. Also, the lattice parameter was calculated using the principal planes corresponding to the Ni-Cr-Fe system (FCC structure); the values correspond with the results for Inconel 600 and Inconel 690 reported by Raju et al. (2004) and Lerot et al. (2001) [45,46], respectively. Based on the former findings, it can be assumed that Cr in solid solution does not affect the atomic spacing; therefore, no additional analyses were performed in this work; the effects of Cr content on H diffusivity were focused on their microstructural influence. On the other hand, the dendritic microstructures of the experimental superalloys and their corresponding XRD spectra are shown in Figure 4. The resulting evidence of OM and XRD are in agreement with the reported literature, showing the main phases: γ and M_7_C_3_ [38]. The most useful XRD spectrum obtained to identify the phases is presented in Figure 4b, corresponding to dendritic samples of the 15Cr alloy. Regarding the micrographs and XRD spectra, it is possible to observe similar phases; however, in some XRD analyses, the M_7_C_3_ precipitates identification was not possible. Despite the above-mentioned phenomenon, due to the main Ni-Cr-Fe-C system, the phases are present in the whole superalloys. Additionally, the crystallite size (T_c_) was calculated by means of diffraction results, applying Scherrer’s equation (T_c_ = Kλ/βcosθ). The values of the variables were extracted from the XRD-characterized information, and a value of 0.9 for the K constant was considered. The average result of the three experimental alloys was 3.36 Å.

### 3.2. Metallography

**Figure 5:**
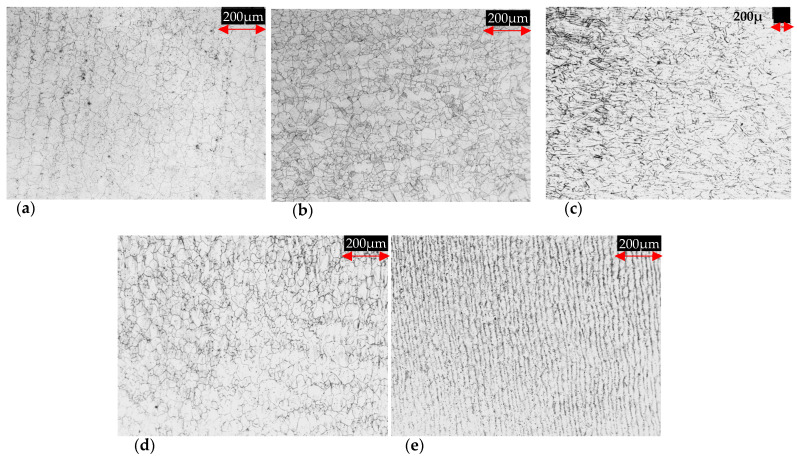
Microstructures of commercial and hot-rolled experimental superalloys: (**a**) In 690, (**b**) In 600, (**c**) 28Cr, (**d**) 15Cr, and (**e**) 10Cr.

In Figure 5a,b, the microstructures of commercial Inconel are shown; their appearance is equiaxed, typical of austenitic alloys. Regarding the experimental alloys, they exhibited microstructures with different morphologies, being generally semi-equiaxed (see Figure 5c–e). The last microstructure may be attained due to the recovery and recrystallization process during the hot rolling, and in addition, due to the potential pinning on grain boundary migration, induced by precipitation. Based on the reported literature, it is possible to consider that the performance of this type of deformed microstructures under H flux conditions can be strongly influenced by grain disorientation related to the high-angle grain boundaries and percolation [47,48]. These studies are outside the scope of this study.

**Figure 6:**
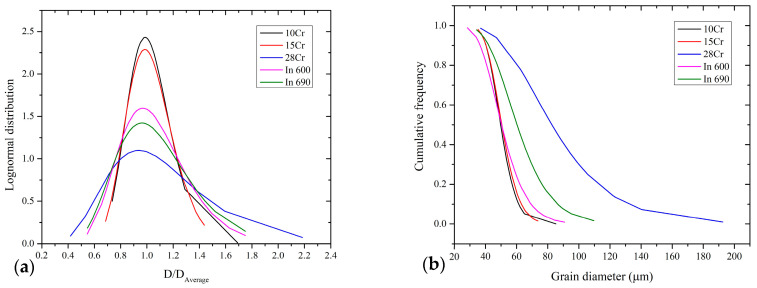
Analysis of grain sizes distribution. (**a**) Grain distribution density, normalized as a function of D, and (**b**) negative cumulative distribution.

The measured features described in Section 2.2 were utilized to perform a microstructures analysis; this was achieved based on the lognormal function and Hillert’s 2D criteria [49], where D = D_grain_/D_average_ was the comparison of each grain diameter over the average grain diameter from all measurements. In addition, abnormality percentages were calculated using the following expression: %abnormality = L_abnormal_/L_total_, where L_abnormal_ represents the sum of grains radius > 2D and L_total_ is the total sum of the grains’ radius. However, just the 28Cr alloy presented abnormality, having 9%; the rest of the material did not show abnormality based on their D value and Hillert’s criteria. Figure 6a shows the calculated behavior of the grain size distribution density for each alloy; it can be appreciated that the 28Cr exhibits values ≥ 2D. On the other hand, the negative cumulative distribution vs. grain size in μm is shown in Figure 6b. Similar grain sizes between In 600, 10Cr, and 15Cr alloys can be observed. The In 690 and 28Cr showed larger sizes, and the last image presents a highly deformed microstructure, observed with OM (see Figure 5c). The measured microstructural characteristics are shown in Table 4. The PF values of the commercial superalloys are higher than the experimental results. This may be caused by the supplier’s thermomechanical process, generating greater precipitation distribution. On the other hand, the APS values for the two Inconel alloys are similar considering the ±value. The experimental superalloys exhibited a slight increase in APS as a function of the Cr addition, and perhaps, this increase in percentage caused greater nucleation, forming larger precipitates.

**Figure 7:**
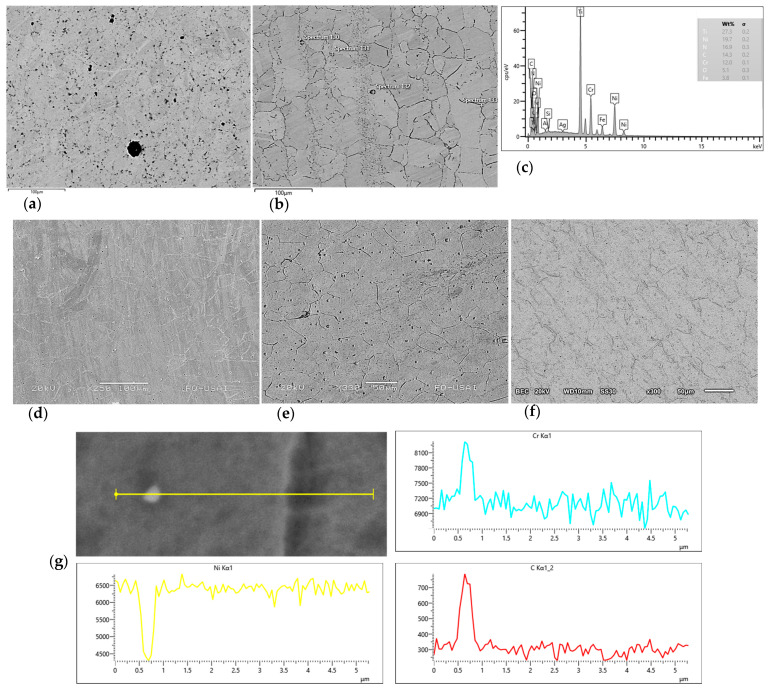
Microstructures of Inconel and hot-rolled experimental superalloys observed with SEM: (**a**) In 690, (**b**) In 600, (**c**) In 690-EDS spectrum, (**d**) 28Cr, (**e**) 15Cr, (**f**) 10Cr, and (**g**) 28Cr-EDS spectrum.

From the SEM analysis conducted, the morphologies of the commercial and experimental hot-rolled microstructures were examined (see Figure 7); grains, grain boundaries, and second-phase particles were observed. Similar to the OM observations, the experimental alloys showed equiaxed microstructures in Inconel and deformed grains. In addition to the morphology study, EDS analysis was performed to obtain qualitative information of the elements present in the matrix and detect possible precipitates. Ti signal was detected for In 600 and In 690, which may indicate the presence of Ti-rich precipitates despite the supplier’s chemical composition report for In 600. On the matrix, the Ni-Cr-Fe system was confirmed for both commercial and experimental materials, with the presence of other elements, added in low proportion in the commercial alloys, as showed in Figure 7c. Regarding the experimental alloys, their main chemical composition is shown in Table 1; nevertheless, EDS line scan analysis was performed. Accordingly, similar compounds were found in the three experimental alloys, as can be seen in Figure 7g (28Cr), indicating Cr and C rich zones, depleted in Ni, and are clearly observed; this can be related to M_7_C_3_ or M_23_C_6_ precipitates.

### 3.3. Permeation Tests

The summary results for D_eff_ and J_ss_ are shown in Table 5. The trapping effect of H on the 10Cr, 28Cr, and In 690 alloys can be seen; this is exhibited by the reduction in the D_eff_ value at the second transient. Furthermore, the J_ss_ also decreases, and the results for 15Cr are included. From this behavior, the mass transfer rate decay, possibly generated by the effect of reversible and irreversible traps, brings about an accumulation within the material during the permeability tests. It is essential to notice that the D_eff_ values obtained for In 600 and In 690 were similar to the presented experimental alloys. The In 690 showed close D_eff_ values in the two transients; the J_ss_ was not affected by the transition between tests. Finally, the J_ss_ of the In 600 in its two conditions presented the highest values, a trend comparable to the first study reported [5]. The former indicates that the trapping effect is even lower due to a high value of molar flux per unit area per time. Remarkably, the 15Cr showed a particular behavior, and the understanding and hypothesis of the reason for this phenomenon are complicated. Initially, from the difference in D_eff_ values, it was likely that there was an increment from the first to the second transient, which may indicate that the saturation by reversible and irreversible traps was not inhibiting the H diffusivity. This effect is possibly related to the microstructure’s state; perhaps, the so-called “percolation” effect is much more significant than the trapping density present. Additionally, the J_ss_ value in this alloy was decreased between transients. Nevertheless, the above-stated phenomenon may depend on several factors, such as sample thickness, chemistry, and microstructure defects, among others; this could be related to the trapping effect, possibly generating H accumulation in the matrix. Regarding the utilized Laplace and Fourier methods, the values calculated did not drastically change compared to the time lag method (approximately a maximum error of 5%).

**Figure 8:**
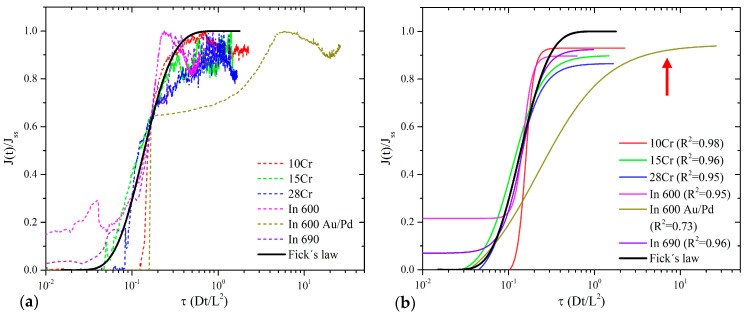
Comparison between J(t)/J_ss_ vs. τ results of the superalloys at the first transient. (**a**) Experimental curves and (**b**) fitted curves. The Fick’s law was plotted as a black line for comparison.

To conclude the analysis of the results, the normalized flux values (J(t)/J_ss_) vs. the normalized time (τ = Dt/L^2^) were plotted for the first transient. A specific solution to Fick’s second law, stipulated in ISO 17081 (Equation 5), was employed, assessing an FCC lattice of pure Ni with D_L_ = 2.31 × 10^−14^ m^2^/s [50]. It is possible to observe from Figure 8 that the alloys results, except for In 600 Au/Pd coated, show compliance with the Fick’s law solution. In agreement with ISO 17081, the H trapping is not significant. On the other hand, due to the behavior presented in the In 600 alloy Au/Pd coated, it is suitable to consider a moderately significant trapping effect (see arrow in Figure 8); however, evaluating its second transient was impossible. The normalized flux results are shown below, as well as their fitted interpretation for better comprehension, in Figure 8b.

Finally, applying Equation (9) [32,33,34,35], the trap density (N_T_) was calculated. The used parameters values were D_eff_ extracted from the experimental data, D_L_ = 2.31 × 10^−14^ m^2^/s, Eb = 0.2 eV [50], and the K temperature and R gas constant were considered. On the other hand, the variable N_L_ represents the interstitial sites density, in this case, related to the Ni-Cr-Fe-C system, considering 12 interstitial sites, 8 tetrahedral and 4 octahedral, corresponding to the FCC structure. For the N_L_ calculation, the equation described by A.H.M. Krom et al. (1999 and 2000) [51] was used (Equation (10)), and in addition, from a developed method in this work, Equation (11) is proposed.

(9) $$\ln\frac{N_{T}}{N_{L}}+\frac{E_{b}}{RT}=\ln\left(\frac{D_{L}}{D_{eff}}-1\right)$$

(10) $$N_{L}=\frac{N_{A}\beta \rho }{PA}$$

(11) $$N_{L}=\frac{{NI}_{C}}{a^{3}}$$

For Equation (10), N_A_ is Avogadro’s number, β represents the number of interstitial sites per atom (three for FCC structures), ρ is the metal density (kg/m^3^), and PA is the atomic weight (kg/mol). Regarding the model developed concerning Equation (11), the number of interstitial sites per unit cell was defined as NI_C_, corresponding to eight tetrahedral and four octahedral sites. The value of a^3^ corresponds to the volume of the unit cell and was obtained using the experimental XRD results. Therefore, considering the resulting values, the order of magnitude achieved for N_T_ was ≈7E25 traps/m^3^; this agrees with the reported percolated microstructures [47,48]. Table 6 summarizes the results obtained for In 600 and 10Cr alloys, which exhibited the highest and the lowest values, respectively. Relating the D_eff_ and trap density results, it can be considered that the trapping is not significant; nevertheless, it can relate the diffusivity behavior with the trapping effect of the analyzed samples.

### 3.4. Predictive Model

**Figure 9:**
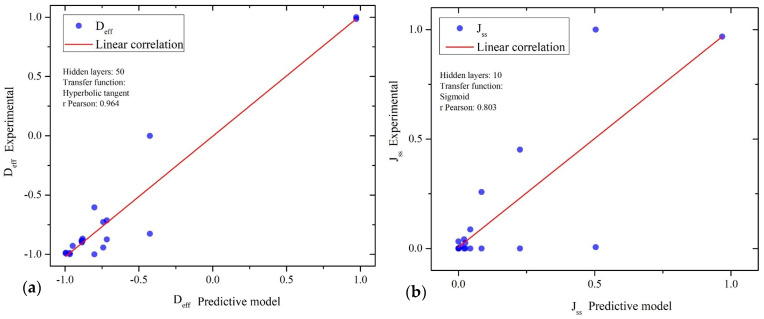
ANNs results: (**a**) effective diffusion coefficient and (**b**) steady-state flux.

Table 7 shows the linear correlation results of the trained models. The predictive modeling for D_eff_ presented better linear correlation than those developed for J_ss_. This may be caused by the behavior of J_ss_ and D_eff_ obtained from data analysis; this confirms that predicting D_eff_ variable is more attainable. The correlation coefficients are acceptable for modelling; therefore, the ANNs can estimate D_eff_ and J_ss_ based on the considered 10 inputs. Once all the correlation coefficients were obtained, the best models trained were chosen as a final proposal for predictive modeling and for its use in the relative importance calculation. In this case, for the D_eff_ prediction, the ANN comprising 50 neurons in the hidden layer and hyperbolic transfer function was selected. Furthermore, for the J_ss_, the ANN selected comprised 10 neurons in the hidden layer and sigmoid transfer function; the resulting models are shown in Table 7, highlighted in green. Figure 9 graphically indicates the data dispersion and linear correlation of the selected models.

**Figure 10:**
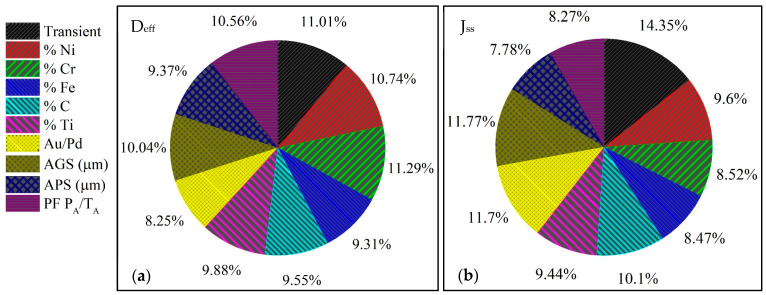
Relatively important results: (**a**) effective diffusion coefficient and (**b**) steady-state flux.

On the other hand, employing the trained ANNs corresponding to D_eff_ and J_ss_, the Garson’s algorithm was used by applying Equation (8). For this, the weights of the ANNs were extracted and the results were expressed in pie charts, shown in Figure 10. From the results obtained, it is possible to comment the following two points in relation to each output variable.

The three input variables with the most significant impact on D_eff_ are the % Cr, number of transient, and the % Ni. The transient significantly impacts the output signal, possibly due to the trapping effect from the first transient to the second. In the case of importance of % Cr and % Ni, these elements could be concatenated due to their balance dependence on % Ni as a function of % Cr. Therefore, another possible variable worth considering is the PF, and its influence is suitable due to the trapping effects caused by precipitation.

The results for J_ss_ suggest the transient as the variable with the most significant importance, and this agrees with the experimental results obtained, as can be seen in Table 5. Subsequently, the Au-Pd coating and the AGS appear in the same magnitude of importance. The Au-Pd coating is uniquely associated with the In 600 alloy; perhaps the coating impacts the J_ss_ by modifying the response at the cathodic and anodic sides of the electrochemical cell. Its influence can be considerable since the experimental J_ss_ values acquired for the In 600 Au/Pd alloys are the highest (see Table 5). Furthermore, Figure 8 shows a displacement concerning τ, suggesting a slight increase in the trapping compared to the rest of the alloys. This effect is exclusively observed in this alloy; therefore, there is an apparent agreement between the experimental evidence and the relative importance calculus. Finally, the importance of AGS is due to the grain size and the number of grain boundaries affecting the H diffusion; this has been reported in the literature [47,48]. The former statement must be complemented with research on grain boundaries and their influence on H diffusivity.

## 4. Discussion

### 4.1. Impact Parameters on the H Diffusivity in Ni-Based Alloys

The main objective of this and the previously reported work [5] was to analyze the Cr effect on the H diffusivity. It was found that in solid solution does not significantly affect interatomic spacing or diffusivity. However, it is possible to consider its effect due to precipitation since, as reported in the literature, the M_7_C_3_ and M_23_C_6_ precipitates act as high-energy traps, having more significant impact than grain boundaries and dislocations [52]. Therefore, an increase in Cr content is mainly impacted by precipitation. Thus, and based on recent research, the main preponderant factor on the H diffusion is related to the microstructural characteristic distribution. Furthermore, assessing the effect of grain size, precipitate fraction, and type of precipitates, it is possible to consider the grain boundaries’ misorientation as the principal impact parameter. In recent years, microstructures of Ni-based alloys have been studied under the misorientation framework and their effects on diffusivity. The work reported by Oudris et al. (2012) [47,48] indicates the relationship between the grain boundaries’ misorientation effect on H diffusion. Additionally, they indicate orders of magnitude for D_eff_ and N_T_ corresponding to percolated grain boundaries, comparable to the findings in this work. Thus, high-angle grain boundaries with random connectivity are more susceptible to H diffusion. In contrast, the Σ3^n^ and low-angle (Σ1) type microstructures have shown trapping and diffusion-obstructing properties. Several strategies, including applying thermomechanical cycles to these alloys, have been developed to induce orientation to the microstructures, achieving particular grain boundaries [53,54,55]. Therefore, setting the objective of avoiding HE, it is necessary to consider the following points:

- High *fΣ* fraction microstructure: It is necessary to consider that the H trapping could be higher in alloys composed of Σ3^n^ and Σ1 grain boundaries. This can be a valuable strategy to avoid H diffusion from the surface and subsurface. However, it would not be possible to avoid it entirely. Consequently, the H would be trapped, triggering some HE mechanism.
- Percolated microstructure: For this case, the literature reports show high H diffusivity since the effect of high-angle grain boundaries and random connectivity at triple junctions considerably accelerate the absorption and diffusion. A percolated microstructure would present greater D_eff_ values, similar to the present work. This percolated effect intends to exhibit a superimposing effect on irreversible traps, even though these traps are preferably formed at high-angle grain boundaries and high-energy triple junctions.

The strategy followed by the authors results in the application of grain boundary engineering. This is for forming Σ3^n^ boundaries, dislocation networks, and twinning. This consideration is due to the adverse effects that the misoriented microstructure can exhibit, associated with stress corrosion cracking and high formation of rich Cr:C precipitates. Consequently, high-energy irreversible trapping sites induce susceptible areas in HE, resulting in a Cr-depleted passive layer.

Based on the results reported in the present work, it is necessary to continue research on the microstructural framework.

### 4.2. Future Considerations in Predictive Modeling Using AI

The predictive modeling applied in this work demonstrates a viable alternative for the D_eff_ calculation and the analysis of the main impact factors. Certainly, the model presented is not complete because of the limitation of the microstructural characterization, samples used in as-received condition, and evaluations of the H permeability test based on the electrochemical double cell. It is necessary to enrich the database with studies focused on different microstructural states (thermomechanical process) and electron back scattering diffraction (EBSD) characterization. In addition, thermal desorption spectroscopy (TDS) is fundamental for a robust understanding of H trapping and diffusion. Regarding the predictive model, it is feasible to improve its performance by evaluating several types of AI learning techniques such as support vectors machines, Bayesian ANN, and others.

## 5. Conclusions

An outline of the present work is as follows. Three alloys were designed and manufactured based on the Ni-Cr-Fe-C system, varying the ranges of Cr content from 10 to 28% wt; this was in order to compare experimental alloys with commercial alloys, In 600 and In 690. Using XRD, the presence of the main phases was confirmed, and their lattice parameters were also calculated. Microstructural characterization was performed, obtaining measurements of AGS, APS, and PF. On the other hand, H permeability tests assessed commercial and experimental Ni-based alloys and Au/Pd-coated In 600. Finally, a predictive model was developed using artificial neural networks, considering 10 input variables; later, the relative importance calculation was carried out utilizing Garson’s algorithm, and this was performed to obtain D_eff_ and J_ss_. The results obtained can point out the following conclusions:

- The increment in the Cr content from 10 to 28% does not cause significant changes in the FCC lattice’s interatomic spacing. The lattice parameters calculated for experimental superalloys are in agreement with literature reports of In 600 and In 690.
- The grain size of the 28Cr alloy differs from the rest of the obtained measurements; nevertheless, based on the permeability results, this difference does not seem to have significant influence on the H diffusivity.
- The 10Cr, 28Cr, and In 690 alloys present a trapping effect, reflected in the D_eff_ results, and exhibit an H accumulation; a discrete decrease in the J_ss_ values validates the latter phenomenon. Regarding the commercial alloys, In 690 exhibits a minimum change in the J_ss_ values between transients. The alloy In 600 showed the highest J_ss_, a behavior that the authors have previously reported. Finally, an increase in D_eff_ and a decrease in J_ss_ were observed for the 15Cr alloy, related to the transients; this effect is possibly generated by reversible trapping, which results in H accumulation.
- From Figure 8, the first transient suggests insignificant H trapping, except for the In 600 Au/Pd alloy.
- An alternative method for N_L_ calculations was proposed, having an approach similar to previous models.
- It was possible to develop predictive models to calculate D_eff_ and J_ss_ based on 10 input variables. The correlation coefficients showed the excellent performance of this AI-based method. The modeling using artificial neural networks can be improved, including additional parameters such as grain boundary misorientation, thermal desorption spectroscopy assessments, and heat treatment application.
- The relative importance calculation presented interesting insights related to the observations and the interpretations of the different results. In general, the transient effect, the elemental content of Ni and Cr, and the Au/Pd coating were the main impact variables on the diffusivity and permeability of H.

## Figures and Tables

**Table 1:** Chemical composition of Inconel and experimental alloys.

Alloy		Chemical Content (% wt.)		
	Cr	Fe	Ni	C
28Cr	28.2	9.3	62.3	0.47
15Cr	15.0	9.6	75.3	0.16
10Cr	10.2	9.3	80.4	0.155
In 690	27.5	9.2	61.6	0.235
In 600	15.7	6.9	77.0	0.325

**Table 2:** Input and output variables for the training process of the predictive models.

Inputs	Outputs
# Number of transient	
Ni % wt	
Cr % wt	
Fe % wt	
C % wt	D_eff_ (m^2^/s)
Ti % wt	J_ss_ (mol/cm^2^s)
Coating Au/Pd	
AGS (µm)	
APS (µm)	
PF (P_A_/T_A_)	

**Table 3:** XRD results.

	Interplanar Spacing, *d*(Å)					
(h k l)	*2-Theta*	10Cr	*2-Theta*	15Cr	*2-Theta*	28Cr
(1 1 1)	44.16	2.049	44.18	2.048	43.78	2.063
(2 0 0)	51.46	1.774	51.46	1.774	51.00	1.787
(2 2 0)	75.761	1.254	75.76	1.254	75.10	1.262
(3 1 1)	92.14	1.069	92.10	1.069	91.32	1.077
	Lattice parameter, *a*(Å)					
(1 1 1)		3.549		3.547		3.573
(2 0 0)		3.548		3.548		3.575
(2 2 0)		3.548		3.548		3.571
(3 1 1)		3.547		3.548		3.574

**Table 4:** Measurement results of the microstructures.

Alloy	Microstructural Characteristic		
	AGD (µm)	APS (µm)	PF P_A_/T_A_
In 690	62.6 ± 17.4	8.0 ± 1.6	1.2 × 10^−3^
In 600	51.9 ± 13.3	9.5 ± 2.1	1.7 × 10^−3^
28Cr	88.1 ± 33.1	7.2 ± 1.3	4.9 × 10^−4^
15Cr	51.2 ± 9.1	5.1 ± 1.0	2.4 × 10^−4^
10Cr	50.4 ± 8.8	6.1 ± 1.4	3.6 × 10^−4^

**Table 5:** Hydrogen permeation tests results, D_eff,_ and J_ss_.

	Effective Diffusion Coefficient (D_eff_, m^2^/s)					
First Transient	10Cr	15Cr	28Cr	In 600	In 600 (Au/Pd)	In 690
t_lag_	1.389 × 10^−8^	7.645 × 10^−10^	2.013 × 10^−9^	1.348 × 10^−11^	7.143 × 10^−10^	2.778 × 10^−9^
Fourier	1.442 × 10^−8^	7.848 × 10^−10^	2.075 × 10^−9^	1.382 × 10^−11^	7.470 × 10^−10^	2.791 × 10^−9^
LaPlace	1.435 × 10^−8^	7.815 × 10^−10^	2.066 × 10^−9^	1.376 × 10^−11^	7.440 × 10^−10^	2.779 × 10^−9^
Second transient	10Cr	15Cr	28Cr	In 600	In 600 (Au/Pd)	In 690
t_lag_	1.225 × 10^−9^	1.913 × 10^−9^	9.311 × 10^−10^			3.087 × 10^−10^
Fourier	1.268 × 10^−9^	1.970 × 10^−9^	9.548 × 10^−10^			3.019 × 10^−10^
LaPlace	1.262 × 10^−9^	1.962 × 10^−9^	9.508 × 10^−10^			3.005 × 10^−10^
	Steady-state flux (J_ss_, mol/cm^2^s)					
First transient	1.09 × 10^−6^	2.04 × 10^−7^	2.12 × 10^−7^	1.93 × 10^−5^	3.19 × 10^−5^	9.77 × 10^−7^
Second transient	6.36 × 10^−7^	1.35 × 10^−7^	1.40 × 10^−7^	---	---	9.97 × 10^−7^

**Table 6:** Trap density results.

Hydrogen Trap Density (Traps/m^3^)			
D_eff_ utilized from the t_lag_ method	10Cr	In 600	Average (all alloys)
	7.1 × 10^25^	7.15 × 10^25^	7.1 × 10^25^

**Table 7:** Results of the linear correlations; the ANNs utilized for the relative importance calculus are marked in green.

Linear Correlation, r Pearson				Neurons in Hidden Layer
D_eff_ (m^2^/s)		J_ss_ (mol/cm^2^s)		
Sigmoid	Hyperbolic	Sigmoid	Hyperbolic	
0.96	0.95	0.80	0.79	10
0.96	0.95	0.79	0.73	20
0.96	0.94	0.79	0.80	30
0.95	0.95	0.67	0.77	40
0.94	0.96	0.74	0.79	50
0.96	0.94	0.79	0.79	60
0.96	0.96	0.73	0.80	70
0.96	0.96	0.79	0.80	80
0.83	0.95	0.80	0.78	90
0.87	0.94	0.72	0.79	100
